# The Role of Cytokine PF4 in the Antiviral Immune Response of Shrimp

**DOI:** 10.1371/journal.pone.0162954

**Published:** 2016-09-15

**Authors:** Yulei Chen, Jiao Cao, Xiaobo Zhang

**Affiliations:** College of Life Sciences, Zhejiang University, Hangzhou, 310058, The People’s Republic of China; Institute of Oceanology, Chinese Academy of Sciences, CHINA

## Abstract

During viral infection in vertebrates, cytokines play important roles in the host defense against the virus. However, the function of cytokines in invertebrates has not been well characterized. In this study, shrimp cytokines involved in viral infection were screened using a cytokine antibody microarray. The results showed that three cytokines, the Fas receptor (Fas), platelet factor 4 (PF4) and interleukin-22 (IL-22), were significantly upregulated in the white spot syndrome virus (WSSV)-challenged shrimp, suggesting that these cytokines played positive regulatory roles in the immune response of shrimp against the virus. Further experiments revealed that PF4 had positive effects on the antiviral immunity of shrimp by enhancing the shrimp phagocytic activity and inhibiting the apoptotic activity of virus-infected hemocytes. Therefore, our study presented a novel mechanism of cytokines in the innate immunity of invertebrates.

## Introduction

Shrimp, one of the most important species in aquaculture, is affected worldwide by diseases, notably those caused by white spot syndrome virus (WSSV). WSSV has resulted in large economic losses of the shrimp aquaculture industry. Therefore, the control of this virus is important to ensure the long-term survival of shrimp aquaculture. Due to the extreme virulence of WSSV, preventing and inhibiting the spread of the virus is very difficult. It is well known that the disease resistance of shrimp, as an invertebrate, is entirely dependent on the innate immune system, including cellular and humoral responses [1]. The innate immune system is the first line of inducible host defense against bacterial, fungal and viral pathogens [2]. Although most of shrimp die because of the WSSV infection, some of the WSSV-infected shrimp survive, indicating that shrimp possess immune factors responsible for the shrimp resistance against the virus invasion. As reported, some shrimp proteins, such as PmAV, hemocyanin, Ran and Rab6, take great effects on the antiviral immunity of shrimp [3–6]. The Toll, immune deficiency (IMD) and Janus kinase/signal transducer and activator of transcription (JAK/STAT) pathways are the primary signaling pathways that regulate the immune response of invertebrates against the virus infection in shrimp [7]. In recent years, small interfering RNAs (siRNAs) and microRNAs (miRNAs) have been found to mediate the antiviral defense in shrimp [8–11]. The siRNAs and miRNAs function by targeting the host and/or virus genes. Up to date, however, the immune factors involved in shrimp defenses against the virus invasion have not intensively investigated.

As well known, cytokines play important roles in the animal immune defenses against pathogenic infection [12, 13]. Generally, cytokines are polypeptides or proteins with low molecular masses that are secreted by activated immunocytes or matrix cells. Cytokines have enormous impacts on the development of the immune system, the host defense and tumor immunobiology [14]. In vertebrates, the innate immune cells, including macrophages and dendritic cells, express Toll-like receptors (TLRs), which bind to conserved sequences expressed by microorganisms [15]. Upon recognition of their ligands on microorganisms, TLRs induce the expression of a variety of host defense genes, including antimicrobial peptides, inflammatory cytokines and chemokines and other effectors against the invading pathogens. The intracellular signaling pathway activated by TLRs is conserved from *Drosophila* to mammals [15]. For viral infections, virus-associated molecules, such as genomic DNA or RNA, produced in infected cells can be recognized by the host pattern-recognition receptors (PRRs) expressed in innate immune cells [16]. After recognition of viral components, PRRs initiate effective antiviral responses in the host, including the production of a variety of cytokines and the induction of inflammatory and adaptive immune responses [17]. Particularly, type I interferon is the key cytokines produced by hosts against the virus infection, which mediate the induction of both the innate immune response and the adaptive immune response to viruses [18,19]. At present, the roles of cytokines in the immunity of vertebrates have been well documented. In invertebrates, several studies have shown that cytokines are present and have various roles, such as the cytokine TNF in the Toll pathways of fruitfly and penaeidin of shrimp [20, 21]. However, the information on the effects of cytokines in the innate immunity of invertebrates is limited.

In this investigation, the cytokines of shrimp were characterized to elucidate the roles of cytokines in the invertebrate immune response against viral infection. The results showed that the cytokine PF4 played an important role in the antiviral immunity of shrimp. Therefore, our study presented a novel aspect of cytokines.

## Materials and Methods

### Shrimp culture and WSSV infection

*Marsupenaeus japonicus* shrimp, approximately 10 g and 5–7 cm each, were purchased in Jinjiang aquaculture market (Hangzhou, China) and cultured in groups of 20 individuals in 80 L aquariums at 20–25°C. Prior to experimental infection by WSSV, the hemolymph of randomly selected shrimp was subjected to PCR detection with WSSV-specific primers (5’-TTGGTTTCATGCCCGAGATT-3’ and 5’-CCTTGGTCAGCCCCTTGA-3’) to ensure that the specimens were virus-free [22]. The virus-free shrimp were infected with 0.1 ml of WSSV virus solution (10^5^ genome copies/ml) by intramuscular injection. Phosphate-buffered saline (PBS) was used as a control. At different times post-infection, the shrimp were collected for later use.

### Acellular hemolymph analysis using a multiplex cytokine antibody array

The shrimp hemolymph was centrifuged at 500×g for 20 min to remove the hemocytes and tissue debris. Subsequently, the acellular hemolymph was subjected to a human cytokine antibody array (G series 4000) from RayBiotech (USA) to detect the cytokine levels. Many cytokines are conserved in animals. In this study, therefore, the human cytokine antibody array was used to screen the shrimp cytokines. This multiplex array could simultaneously measure 274 different cytokines on a glass chip. Briefly, cytokine array membranes were blocked with 5% BSA (bovine serum albumin)/TBS (0.01 M Tris-HCl, 0.15 M NaCl, pH 7.6) for 1 h. The membranes were then incubated with the samples after normalization with equal amounts of proteins. After extensive washing with TBS/0.1% (v/v) Tween 20 and TBS to remove unbound materials, the membranes were incubated with a cocktail of biotin-labeled antibodies against different individual cytokines. The membranes were then washed and incubated with HRP (horseradish peroxidase)-conjugated streptavidin (2.5 pg/ml) for 1 h at room temperature. Unbound HRP-streptavidin was washed away with TBS/0.1% Tween 20 and TBS [23, 24]. Finally, detection was performed with a chemiluminescence imaging system (Bio-Rad, Hercules, CA, USA), and images were quantified using Quantity One (Bio-Rad, USA), followed by normalization using the RayBiotech analysis tool (RayBiotech, GA, USA).

### Western blot analysis

The shrimp hemocytes were lysed in RIPA lysis buffer (Beyotime, China) containing 2 mM phenylmethanesulfonyl fluoride (PMSF) on ice. The protein concentration was determined using a BCA protein assay kit (Beyotime Institute of Biotechnology, Shanghai, China), and 20 μg of protein was used for each sample. After separation on a 15% SDS-polyacrylamide gel, the proteins were electrotransferred to a nitrocellulose membrane (GE Healthcare, Germany) in transfer buffer (25 mM Tris, 190 mM glycine, 20% methanol). The membrane was blocked with 5% nonfat milk in TBST buffer (20 mM Tris-HCl, 150 mM NaCl, 0.05% Tween 20, pH 8.0) for 2 h at room temperature. Subsequently, the membrane was incubated with a primary antibody in TBST buffer containing 1% nonfat milk overnight at 4°C. After extensive washing in TBST buffer, the membrane was incubated with horseradish peroxidase-conjugated secondary antibody (Bio-Rad, USA) for 2 h at room temperature. The membrane was detected by using a Western Lightning Plus-ECL kit (Perkin Elmer, USA). The monoclonal antibody against Fas was purchased from Cell Signaling Technology (USA). The polyclonal antibodies against PF4 and IL-22 were purchased from Abcam (USA).

### Evaluation of cytokines in the viral infection of shrimp

The virus-free shrimp were injected with 0.1 ml of WSSV virus solution (10^5^ copies/ml) mixed with cytokines at a concentration of 150 ng/shrimp or 500 ng/shrimp by intramuscular injection [25, 26]. The cytokines were purchased from Peprotech (Rocky Hill, USA). As controls, WSSV alone, cytokines alone and PBS were injected. At different times after injection, the shrimp were collected for later analysis. The cytokine dosage assays showed that 150 ng of cytokines was optimal.

### Detection of WSSV copies by quantitative real-time PCR

Quantitative real-time PCR was performed to measure the WSSV copies in shrimp. The viral DNA was extracted from shrimp gills using a tissue DNA extraction kit (Tiangen, China), and the WSSV copies were detected by real-time PCR with WSSV-specific primers (5’-TTGGTTTCAGCCCGAGATT-3’ and 5’-CCTT GGTCAGCCCCTTGA-3’) and a WSSV-specific TaqMan probe (5’-FAM-TGCTGCCGTCTCCAA-Eclipse-3’) [27]. A linearized plasmid containing a 1400 bp DNA fragment from the WSSV genome was quantified and serially diluted 10-fold as an internal standard for real-time PCR. The 10 μl PCR solution contained 5 μl of Premix Ex Taq (Perfect Real Time) (TaKaRa, Japan), 0.2 μl of 10 μM forward primer, 0.2 μl of 10 μM reverse primer, 0.15 μl of 10 μM TaqMan fluorogenic probe, 200 ng of DNA template, and distilled water up to 10 μl. The real-time PCR conditions were 95°C for 1 min followed by 45 cycles of 30 s at 95°C, 30 s at 52°C, and 30 s at 72°C.

### Shrimp mortality analysis

The virus-free shrimp were intramuscularly injected with 0.1 ml of WSSV virus solution (10^5^ copies/ml) which was mixed with a cytokine. As controls, WSSV alone, cytokine alone and PBS were included in the injections. Twenty shrimp were used for each treatment. The cumulative mortality of shrimp with different treatments was examined at different time points after the injection of WSSV and cytokine (12, 24, 48, and 72 h). The experiments were biologically repeated three times.

### The detection of caspase 3/7 activity

The caspase activity of shrimp hemocytes was detected using a Caspase-Glo^®^ 3/7 assay (Promega, USA) according to the manufacturer’s instructions. Briefly, 100 μl of Caspase-Glo 3/7 reagent was mixed with 4×10^5^ shrimp hemocytes. Then, the mixture was incubated at room temperature for 1 h. The mixture was assayed using a GloMax^®^ 96 Microplate Luminometer (Promega) to detect the caspase 3/7 activity.

### The phagocytosis assay

The purified WSSV virions (10^5^ copies/mL) were treated with 1% paraformaldehyde overnight. After two washes with 0.1 M NaHCO_3_ (pH 9.0), the virions were incubated in 0.1 M NaHCO_3_ containing 1 mg/mL fluorescein isothiocyanate (FITC) (Sigma, USA) for 1 h at 25°C with gentle stirring [28]. After several washes with PBS, the FITC-labeled WSSV virions were incubated with the shrimp hemocytes for 30 min at 28°C, followed by two washes with PBS to remove the unbound FITC-labeled WSSV virions. Subsequently, the hemocytes were resuspended in 1% paraformaldehyde (Sigma, USA) on ice and subjected to flow cytometry (Beckman Coulter, USA) to evaluate the phagocytosis percentage [5].

### Immunoprecipitation (IP) assay

Shrimp hemocytes were lysed in lysis buffer (50 mM Tris-HCl, 150 mM NaCl, 0.5% Triton X-100, pH 7.5) for 1 h on ice. After centrifugation at 12,000×g for 10 min, the supernatant (hemocyte lysate) was collected. Then, an anti-PF4 IgG (Abcam, USA) or isotype IgG (Beyotime, China) was incubated with protein A-coupled Sepharose (GE Healthcare, USA) in lysis buffer for 1 h at 4°C, followed by three washes with lysis buffer. The Sepharose was incubated with the hemocyte lysate overnight at 4°C. Subsequently, the Sepharose was centrifuged at 5,000×g for 5 min and washed three times using lysis buffer. The CoIP products were analyzed by SDS-PAGE with Coomassie brilliant blue staining. The protein band of interest was identified by mass spectrometry.

### Statistical analysis

The numerical data were analyzed using one-way analysis of variance. A t test was conducted to analyze the differences between treatments.

## Results

### Cytokines involved in viral infection

**Fig 1:**
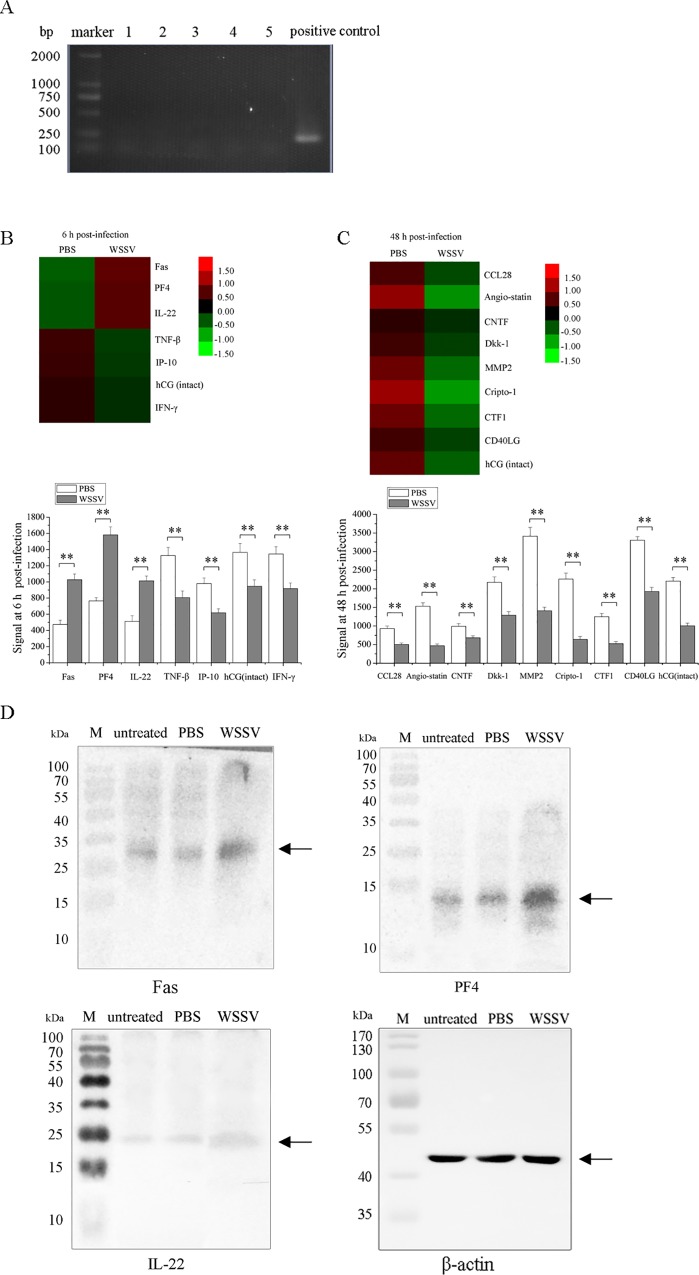
Cytokines involved in viral infection of shrimp. The shrimp were infected with WSSV. At different times post-infection (6 h and 48 h), acellular hemolymph was subjected to cytokine antibody arrays. PBS was used as a control. (**A**) The detection of WSSV in shrimp. Prior to the WSSV infection, PCR was conducted to ensure that the shrimp were virus-free. Numbers indicated representatives of shrimp for the WSSV detection by PCR. (**B**) The shrimp cytokines in response to viral infection at 6 h post-infection. (**C**) The shrimp cytokines in response to viral infection at 48 h post-infection. (**D**) Western blot analysis of the upregulated cytokines in WSSV-challenged shrimp hemocytes at 6 h post-infection. The untreated shrimp (untreated) and PBS were used as controls. Shrimp β-actin served as a loading control. M, protein marker. Statistically significant differences between treatments are represented with asterisks (**, *p*<0.01).

To elucidate the roles of cytokines in viral infection, the cytokines of shrimp infected with WSSV were screened using cytokine antibody arrays. Prior to the infection experiments, polymerase chain reaction (PCR) analysis was conducted to detect the WSSV infection. The results showed that the shrimp used were virus-free (Fig 1A). Based on the conservation of cytokines in various species, the human cytokine antibody array was employed in this study. The results showed that three cytokines, including the Fas receptor (Fas), platelet factor 4 (PF4) and interleukin-22 (IL-22), were significantly upregulated in WSSV-infected shrimp at 6 h post-infection compared with the control (PBS) (Fig 1B), suggesting that these cytokines might play positive regulatory roles in the shrimp immune response against the WSSV infection. At 6 h and 48 h post-infection, several cytokines were significantly downregulated in virus-infected shrimp (Fig 1B and 1C), showing that these cytokines might facilitate the viral infection.

Western blot analysis revealed that the levels of Fas, PF4 and IL-22 were much higher in WSSV-challenged shrimp hemocytes than those in PBS-treated or untreated shrimp hemocytes (Fig 1D). The data of Western blots confirmed the results of the cytokine antibody arrays.

Taken together, the results indicated that the cytokines Fas, PF4 and IL-22 may be involved in the antiviral immunity of shrimp.

### The effect of PF4 on the viral infection in shrimp *in vivo*

**Fig 2:**
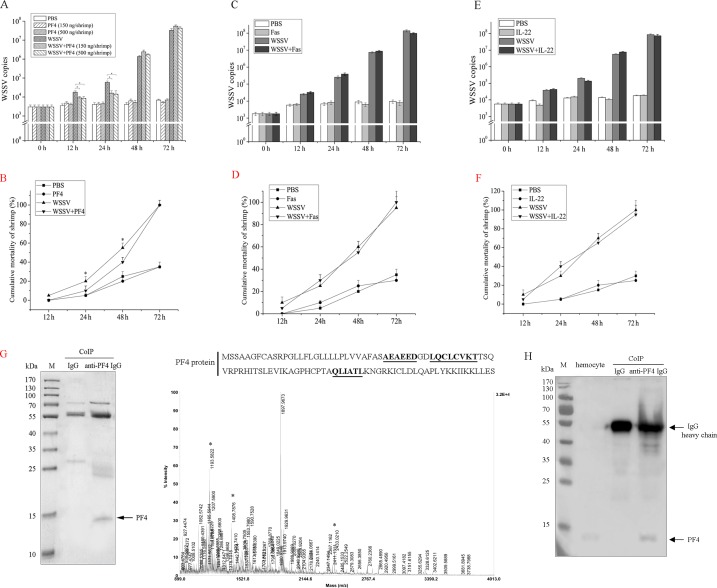
The roles of cytokines in the antiviral immunity of shrimp. (**A**) The influence of PF4 on WSSV copies in shrimp. The shrimp were simultaneously injected with WSSV and PF4 (at various concentrations). At different time points post-infection, the shrimp were subjected to quantitative real-time PCR to quantify the WSSV copies. As controls, PBS, PF4 alone (at various concentrations) and WSSV alone were included in the injections. (**B**) The effects of PF4 on the mortality of WSSV-infected shrimp. The shrimp were simultaneously injected with WSSV and PF4 (150 ng/shrimp). PBS, PF4 alone (at various concentrations) and WSSV alone were included in the injections as controls. At different times after injection, the cumulative mortality of shrimp was monitored. (**C**) The role of Fas in the virus infection of shrimp. The shrimp were simultaneously injected with WSSV and Fas (150 ng/shrimp). At different times post-infection, the WSSV copies of shrimp were quantified by quantitative real-time PCR. As controls, PBS, Fas alone and WSSV alone were included in the injections. The numbers indicated the time points post-infection. (**D**) The influence of Fas on the WSSV-infected shrimp mortality. At different times after injection of WSSV+Fas, the cumulative mortality of shrimp was examined. PBS, Fas alone and WSSV alone were used as controls. (**E**) The impact of IL-22 on WSSV copies in shrimp. The shrimp were treated with WSSV+IL-22 (150 ng/shrimp), PBS, IL-22 alone or WSSV alone, followed by the detection of WSSV copies using quantitative real-time PCR. The numbers indicated the time points post-infection. (**F**) The effects of IL-22 on the mortality of WSSV-infected shrimp. The cumulative mortality of shrimp treated with WSSV+IL-22 (150 ng/shrimp), PBS, IL-22 alone or WSSV alone was evaluated at different times post-infection. **(G)** The identification of PF4 protein in shrimp. Coimmunoprecipitation (CoIP) assays were conducted using shrimp hemocytes with anti-PF4 IgG (left panel). Rabbit IgG was used as a control. The proteins were analyzed using SDS-PAGE with Coomassie brilliant blue staining. Then the protein was identified by mass spectrometry (right panel). The matched peptides were underlined. M, protein marker. **(H)** Western blot detection of PF4 protein in the shrimp hemocytes and in the CoIP products. The proteins in the shrimp hemocytes and in the CoIP products were separated by SDS-PAGE. Then Western blotting was conducted using the antibody against the PF4 protein. The proteins were indicated by arrows. M, protein marker. In all panels, statistically significant differences between treatments are represented with asterisks (*, *p*<0.05).

To explore the effects of the cytokines Fas, PF4 and IL-22 on the viral infection in shrimp, the WSSV-infected shrimp were challenged with the three cytokines. The results showed that the WSSV levels were significantly decreased by PF4 at different concentrations in shrimp infected with WSSV at 12 h to 24 h post-infection compared with the controls (Fig 2A). However, PF4 had no effect on the WSSV infection at 48 h to 72 h post-infection (Fig 2A). At the same time, PF4 alone yielded similar results to PBS (Fig 2A), suggesting that PF4 was not cytotoxic to shrimp. The results indicated that PF4 at different concentrations had similar effects on the viral infection (Fig 2A). Therefore, a PF4 concentration of 150 ng/shrimp was used in the following experiments. The data on the cumulative mortality of the shrimp indicated that PF4 had antiviral activity in WSSV-infected shrimp (Fig 2B). These findings demonstrated that PF4 was involved in the antiviral immunity of shrimp.

The cytokines Fas and IL-22 had no effect on the WSSV copies and the mortality of virus-infected shrimp (Fig 2C, 2D, 2E and 2F), indicating that Fas and IL-22 were not involved in the shrimp immunity.

To confirm the presence of PF4 in shrimp, shrimp hemocytes were subjected to CoIP assays. The results revealed that there existed a specific protein in the CoIP product using the anti-PF4 IgG compared with the control (Fig 2G). Mass spectrometry analysis showed that this protein was identified to be PF4 with 19.8% coverage of amino acid sequence to the known PF4 (Fig 2G), indicating that PF4 was present in shrimp. Western blot analysis revealed that the PF4 protein could be detected in the shrimp hemocytes and in the CoIP products (Fig 2H), showing the presence of the PF4 protein in shrimp.

### The regulation of shrimp apoptosis by PF4

**Fig 3:**
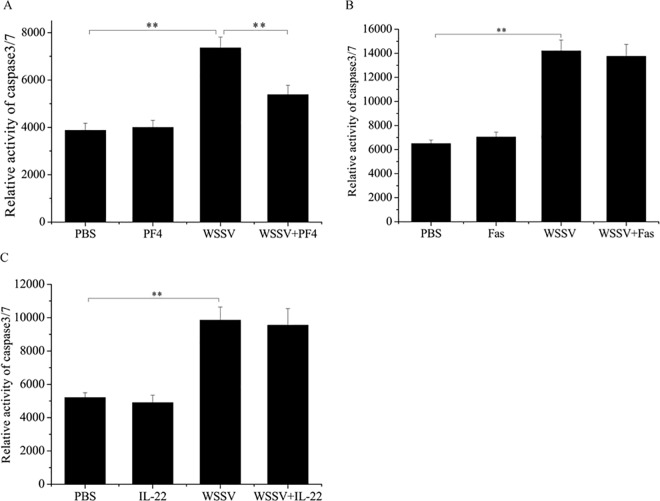
Effects of cytokines on the apoptosis of shrimp hemocytes. Shrimp were simultaneously injected with WSSV and PF4 (**A**), WSSV and Fas (**B**) or WSSV and IL-22 (**C**). Twenty-four hours later, the shrimp hemocytes were subjected to caspase 3/7 activity assays. As controls, PBS, PF4 alone, Fas alone, IL-22 alone and WSSV alone were used in the injections. Data are shown as the mean of three independent experiments. Statistically significant differences between treatments are indicated with asterisks (**, *p*<0.01).

To evaluate the effects of the cytokines Fas, PF4 and IL-22 on shrimp apoptosis, the caspase 3/7 activity of shrimp hemocytes was examined. The results showed that the cytokines alone had no effect on shrimp apoptosis compared with the control PBS (Fig 3A, 3B and 3C). However, WSSV alone resulted in a significant increase in caspase 3/7 activity in shrimp hemocytes compared with PBS (Fig 3A, 3B and 3C), indicating that the virus promoted host apoptosis. The results revealed that the caspase 3/7 activity of shrimp hemocytes treated with WSSV+PF4 was significantly decreased compared with that of WSSV alone (Fig 3A), while Fas and IL-22 had no effect on WSSV-mediated shrimp apoptosis (Fig 3B and 3C). The above data indicated that PF4 played a negative regulatory role in shrimp apoptosis *in vivo*.

### Regulation of phagocytosis in shrimp hemocytes by PF4 *in vivo*

**Fig 4:**
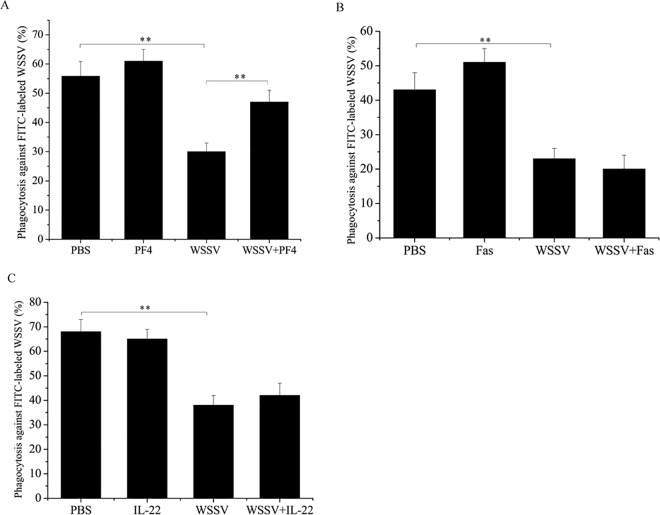
The evaluation of cytokines in the regulation of shrimp phagocytosis against the viral infection. Shrimp were simultaneously injected with WSSV and PF4 (**A**), WSSV and Fas (**B**) or WSSV and IL-22 (**C**). PBS, PF4 alone, Fas alone, Il-22 alone and WSSV alone were included in the injections as controls. At 24 h after the injections, the shrimp hemocytes were subjected to the phagocytosis assay. The phagocytosis percentage was evaluated with flow cytometry. The experiments were biologically repeated three times. Statistically significant differences between treatments are shown with asterisks (**, *p*<0.01).

To characterize the role of the cytokines Fas, PF4 and IL-22 in the regulation of phagocytosis, the phagocytic activity of shrimp hemocytes was evaluated with FITC-labeled WSSV virions. The cytokines alone had no effect on the phagocytic activity of shrimp hemocytes compared with the PBS control (Fig 4A, 4B and 4C). However, WSSV treatment alone led to a significant decrease in the phagocytic activity of shrimp hemocytes (Fig 4A, 4B and 4C). The results showed that the shrimp phagocytic activity was significantly increased with the WSSV+PF4 treatment (Fig 4A), indicating that PF4 played a positive regulatory role in shrimp phagocytosis. However, Fas and IL-22 had no effect on the phagocytosis of shrimp hemocytes against WSSV infection (Fig 4B and 4C). Based on the above findings, we concluded that PF4 was involved in the shrimp phagocytosis against the viral infection.

## Discussion

As a primary seafood for humans, global shrimp production is a flourishing industry [29]. However, diseases caused by WSSV are a limiting factor in shrimp farming. Thus far, there are no efficient strategies for disease control in shrimp, although many studies on protecting shrimp from diseases have been conducted [30–32]. Shrimp defend themselves against pathogens by innate immunity, which includes the humoral immune responses and cellular immune responses, such as phagocytosis and apoptosis [33, 34]. To date, the roles of cytokines in inflammation and immunoregulation are well established. Studies have shown that polymorphisms and mutations in cytokine receptors can lead to autoimmune disorders [12, 35, 36]. Among the cytokines, several chemokines have biologically significant roles in hemostasis/thrombosis in mammals. In recent years, some reports demonstrate that cytokines are present in shrimp, such as vascular endothelial growth factor (VEGF) 1, VEGF2, VEGF3, astakine, penaeidin and interleukin (IL) 1-like protein [37–39]. However, the function of cytokine/chemokine in invertebrates has not been intensively studied. In this study, the results showed that PF4 was involved in the antiviral immune response of shrimp by inhibiting the viral infection *in vivo*. Therefore, our study revealed a novel mechanism of cytokines in shrimp immunity.

PF4, a member of the CXC chemokine subfamily, is synthesized by megakaryocytes and stored in platelet α-granules. In mammalian species, PF4 is expressed at high levels, suggesting that PF4 has important biological activities in mammals [40]. PF4 has been shown to play a pathological role in heparin-induced thrombocytopenia in mice, but the biological functions of PF4 are not well understood. Acceleration of atherosclerosis and the regulation of megakaryopoiesis have also been linked to PF4, as well as the activation of leukocytes, including monocytes, neutrophils and NK cells. Recent studies have shown that PF4 was correlated with angiogenesis and immune responses and was a marker of early tumor growth in different tumor types [41, 42]. In this study, the findings showed that PF4 in shrimp was involved in the defense against the WSSV infection, suggesting a novel function of PF4 in invertebrates. The results indicated that PF4 took effects on the WSSV infection at 12–24 but not 48–72 h post-infection. As reported, the half-life time of the injected PF4 in rabbit is short [43]. Due to the shrimp metabolism, therefore, the injected PF4 might be cleared at 48 h onwards after the PF4 injection in shrimp.

Our study indicated that three cytokines (Fas, PF4 and IL-22) were upregulated in WSSV-infected shrimp at 6 h post-infection. However, only the cytokine PF4 played important roles in the immune responses of shrimp by regulating the phagocytic activity and apoptosis of shrimp hemocytes, thus defending shrimp against the WSSV infection and promoting shrimp survival. During the pathogen invasion, phagocytosis contributes to the first-line response against pathogen infection [44]. Pathogens in the phagosomes are destroyed by intrinsic radical attack and hydrolysis [45]. Our study revealed that PF4 could enhance the hemocytic phagocytosis in shrimp, resulting in an increase of shrimp antiviral immune activity. At the same time, PF4 decreased the apoptotic activity of shrimp during the WSSV infection. Apoptosis is an active process of cell death, and it serves diverse functions in organisms. The induction of apoptosis in virus-infected cells is an important host defense mechanism against invading pathogens [46]. In this study, the results showed that PF4 increased the shrimp phagocytic activity against the virus infection. As a result, the percentage of WSSV-infected shrimp cells were decreased, leading to the reduction of shrimp apoptotic cells and the decrease of shrimp apoptotic activity compared with the positive control (WSSV alone). In mammals, PF4 was found to augment monocyte phagocytosis and is a potent activator of phagocytosis. PF4 not only increases the number of monocytes taking up pathogens but also enhances the amount of pathogens ingested by the cells [47]. PF4 can also prevent circulating monocytes from undergoing apoptosis and facilitate differentiation of monocytes into macrophages during the inflammatory process [48]. In this context, PF4 might enhance the shrimp antiviral immunity by increasing the phagocytic activity of shrimp hemocytes and reducing the apoptosis of circulating hemocytes. Our study found a novel mechanism of PF4 in the regulation of phagocytosis and apoptosis of shrimp. Monocytes treated with PF4 have been reported to generate macrophages with a high capacity for unspecific phagocytosis. Thus, the uptake of pathogens is predominantly processed by unspecific phagocytosis during the initial steps of immune defense in mammals [47]. PF4 may function in the shrimp immune defense in a pathogen-independent manner.
